# Manufacturing of convalescent plasma of COVID-19 patients: Aspects of quality

**DOI:** 10.1371/journal.pone.0243967

**Published:** 2020-12-22

**Authors:** Viola Hähnel, David Peterhoff, Veronika Bäuerlein, Andreas-Michael Brosig, Irene Pamler, Christian Johnson, Adelina Bica, Monica Totir, Thomas Ossner, Barbara Stemmer, Martina Toelge, Anja Schütz, Hans-Helmut Niller, Barbara Schmidt, Ralf Wagner, André Gessner, Ralph Burkhard, Robert Offner

**Affiliations:** 1 Institute of Clinical Chemistry and Laboratory Medicine, Transfusion Medicine, University Hospital Regensburg, Regensburg, Germany; 2 Institute of Medical Microbiology and Hygiene, University Hospital Regensburg, Regensburg, Germany; 3 Institute of Clinical Microbiology and Hygiene, University Hospital Regensburg, Regensburg, Germany; FDA, UNITED STATES

## Abstract

The ongoing coronavirus disease 2019 (COVID-19) pandemic emerged in December 2019. Convalescent plasma represents a promising COVID-19 treatment. Here, we report on the manufacturing of a plasma-based product containing antibodies specific to SARS-CoV-2 obtained from recently recovered COVID-19 patients. Convalescent plasma donors were screened as follows: 1) previously confirmed SARS-CoV-2 infection (by real-time PCR (RT-PCR)); 2) a subsequent negative PCR test followed by a 2-week waiting period; 3) an additional negative PCR test prior to plasmapheresis; and 4) confirmation of the presence of SARS-CoV-2 specific antibodies. Convalescent plasma was stored fresh (2–6°C) for up to 5 days or frozen (-30°C) for long-term storage. Donor peripheral blood and final plasma product were assayed for binding antibodies targeting the SARS-CoV-2 S-protein receptor-binding domain (RBD) and their titers measured by an enzyme-linked immunosorbent assay (ELISA). We performed 72 plasmaphereses resulting in 248 final products. Convalescent plasma contained an RBD-specific antibody titer (IgG) ranging from 1:100 to 1:3200 (median 1:800). The titer was congruent to the titer of the blood (n = 34) before collection (1:100–1:6400, median 1:800). Levels of IL-8 and LBP of donors were slightly increased. Therapeutic products derived from a human origin must undergo rigorous testing to ensure uniform quality and patient safety. Whilst previous publications recommended RBD-specific binding antibody titers of ≥ 1:320, we selected a minimum titer of 1:800 in order to maximize antibody delivery. Production of highly standardized convalescent plasma was safe, feasible and was readily implemented in the treatment of severely ill COVID-19 patients.

## Introduction

The outbreak of the coronavirus disease 2019 (COVID-19) pandemic began in December 2019 in Wuhan, China, and has quickly spread worldwide. Most COVID-19 patients develop mild symptoms including loss of smell, fever and cough. However, in a certain percentage of patients COVID-19 leads to a severe, life-threatening illness [1–4]. Severe acute respiratory syndrome coronavirus 2 (SARS-CoV-2), the causal agent of COVID-19, is an enveloped virus consisting of a positive-sense single-stranded ∼30kb RNA genome [5]. The uptake of the virus occurs via the human angiotensin-converting enzyme-2 (ACE-2) which is expressed on alveolar epithelial cells and endothelium but also in organs such as kidney or intestinal endothelium [6]. One of the mechanisms responsible for the severity of COVID-19 is an extensive release of pro-inflammatory cytokines such as interleukin (IL)-6 or interferon γ [7–9] resulting in a so called “cytokine storm”.

To date, more than ~ 50 million confirmed COVID-19 infections worldwide and more than 1.2 million deaths (November 9^th^ 2020, World Health Organization (WHO) dashboard) have been reported. Patient age and/or the presence of preexisting conditions such as diabetes, obesity or cardiovascular disease have been suggested as factors that can lead to a severe COVID-19 prognosis [10–12].

Except for Remdesivir, there are no approved antiviral agents or vaccines available for the treatment or prevention of COVID-19 infection. Despite a number of high profile clinical trials endorsed by the WHO to examine the effectiveness of existing antiviral therapies including Lopinavir [13] or chloroquine [14] towards COVID-19, results so far have been disappointing.

Convalescent plasma therapy is a classical immunotherapy which was already successfully applied to other infectious diseases such as the Spanish flu, which killed more than 50 million people worldwide between 1917 and 1919 [15], SARS [16] and MERS [17]. The collection of data to assess the efficacy of convalescent plasma in COVID-19 treatment has been hampered by a lack of large-scale, rigorous controlled, randomized clinical trials. Nevertheless, the use of convalescent plasma in patient treatments seems to be a rational therapeutic approach [18, 19].

As of November 2020, there has been a steady rise in the number of case studies published describing the use of convalescent plasma for COVID-19 [20, 21]. Shen et al. reported about critically ill patients with normalized body temperature and decreased virus load after treatment with plasma [22]. In addition, discontinued SARS-CoV-2 shedding and improved outcome have been described [23, 24].

Following a sharp rise in COVID-19 patients admitted to the University Hospital Regensburg, Germany, permission was sought from the local authority (District Government of Upper Franconia) for the emergency production of convalescent plasma. Permission was granted on April 2^nd^ 2020. Between April and June 2020, we obtained plasma from recently recovered COVID-19 patients evaluated for the presence of SARS-CoV-2 specific antibodies. Here, we report on the characterization of plasma products, with reference to product safety and quality. All procedures were performed according to regulatory requirements (Paul-Ehrlich Institute) and in compliance with the German hemotherapy guideline.

## Materials and methods

### Donors

From April to June 2020, plasma was obtained according to the EU Guidelines for Good Manufacturing Practice [25] from 34 donors (68% male) with a median age of 36 years. After initial confirmation of SARS-CoV-2 infection by real-time PCR (RT-PCR), donors were accepted 2 weeks after a negative RT-PCR result. Prior to plasmapheresis, an additional negative PCR and confirmation of SARS-CoV-2 positive antibodies were required. None of the donors underwent intensive care treatment during their COVID-19 illness. Convalescent plasma was manufactured in accordance with the permission of the local authority (District Government of Upper Franconia). The treatment of severely ill COVID-19 patients with convalescent plasma was authorized by the local ethic committee and the Legal Department (University Hospital Regensburg), by the Federal Supervisory Authority Paul-Ehrlich-Institute (PEI) and was conducted according to the principles expressed in the Declaration of Helsinki. All donors provided written informed consent.

### Plasmapheresis

Apheresis was carried out with the Trima Accel Automated Blood Collection System (Terumo BCT) with software version 6.0. 1343–3842 mL of blood volume was processed (median 2859 mL). Up to 800 mL plasma (male) and 600 mL plasma (female) was collected and divided into 200 mL bags/products. Initially, convalescent plasma product was stored 2–6°C for up to 4 days after the day of apheresis. Following a decrease in COVID-19 patients (and in order to create a readily available plasma bank for any future requirement), from May 15^th^ 2020 plasma was frozen within 24 h and stored at -30°C.

### Analytics

#### Antibody titers

Antibodies targeting the SARS-CoV-2 S-protein receptor binding domain (RBD) and their titers (IgG) were measured from peripheral blood of the donor and from the collected plasma using an enzyme-linked immunosorbent assay (ELISA). The optical density was divided by the cut-off to obtain the sample-to-cutoff ratio (S/Co) [26]. To obtain titers, serum and plasma products were tested after respective dilutions (1:100, 1:200, 1:400, 1:800, 1:1,600, 1:3,200, 1:6,400).

#### Cytokines

Cytokines, lipopolysaccharide binding protein (LBP) and bactericidal permeability increasing protein (BPI) of donors`serum were measured as recently described [27] using Luminex® technology (Austin, TX, USA). BPI and LBP levels were determined with an in-house method using specific commercial available antibody pairs (αBPI capture antibody 3F9 and αBPI detection antibody 4H5, Hycult Biotech, Uden, Netherlands¸ αLBP capture antibody biG43 and αLBP detection antibody biG412, Biometec, Greifswald, Germany). Biotinylation of the detection antibodies was performed applying the Lightning-Link® Biotin Conjugation Kit (Innova Biosciences, Cambridge, UK). Cytokines were determined with the commercial ProcartaPlex® Multiplex Immunoassay (eBioscience, Santa Clara, CA, USA). Pre-pandemic control samples obtained from healthy donors (n = 20) were measured for comparison.

#### SARS-CoV-2 NAT

Plasma from first donation of each donor was tested for residual SARS-CoV-2 RNA using the E-gene and RdRp qPCR as described recently [28].

#### Cell count

Cell concentrations were measured undiluted on an XN-550 Automated Hematology Analyzer (Sysmex, Kobe, Japan) per manufacturer's instructions.

#### Sterility testing

Aerobic and anaerobic culture bottles (BD Bactec Standard Anaerobic/F and Aerob/F, respectively) were incubated for seven days at 30–32°C with a sample volume of 6 mL. Sterility testing was performed as previously described [29].

#### Statistics

Since this treatment regime was not part of a pre-planned prospective study, all data were retrospectively analyzed. Microsoft Excel 2010, R (version 3.4.3) and IBM SPSS Statistics 25 were used to collect data and generate figures. Descriptive statistics included the absolute number, frequency, mean/standard deviation, and median/interquartile range. Correlation was determined with the Pearson test. P-values below 0.05 were considered statistically significant.

## Results and discussion

Convalescent plasma was produced in the context of an “individual healing attempt” for COVID-19 patients. For donor selection, we followed the requirements of the local authority and WHO. Before the first plasmapheresis, donors were required to provide two negative SARS-CoV-2 PCR results. Our center started manufacturing of convalescent plasma on April 6^th^ 2020. In total, 34 donors were recruited (68% male) with a median age of 36 years (see Table 1). The majority of the donors exhibited blood groups 0 (n = 14) and A (n = 11), which reflects a typical blood group distribution.

**Table 1:** Donor characteristics.

Number of donors	34
Sex	68% (male), 32% (female)
Age	18–59 years
Blood group	0 (41.2%), A (32.4%), B (14.7%), AB (11.5%)
Number of donations	72
Number of products	248 (153 fresh plasma, 95 frozen plasma)
Frequency of donation	41.2% (1x), 32.4% (2x), 14.7% (3x), 2.9% (4x), 2.9% (5x), 5.9% (6x)

Characteristics of the study population and product numbers are listed.

All donors were tested and found negative for the following infectious agents: HIV, HBV, HCV, HEV and Treponema pallidum, as well as for irregular antibodies against red blood cells on the day of apheresis. To avoid the risk of transfusion-related acute lung injury (TRALI) provoked by the presence of HLA-antibodies, previously pregnant females were excluded from convalescent plasma donation. To protect patient and staff safety, a SARS-CoV-2 PCR was performed on plasma obtained from the first donation. All products showed a negative result and verified the absence of any SARS-CoV-2 RNA in the convalescent plasma.

**Fig 1:**
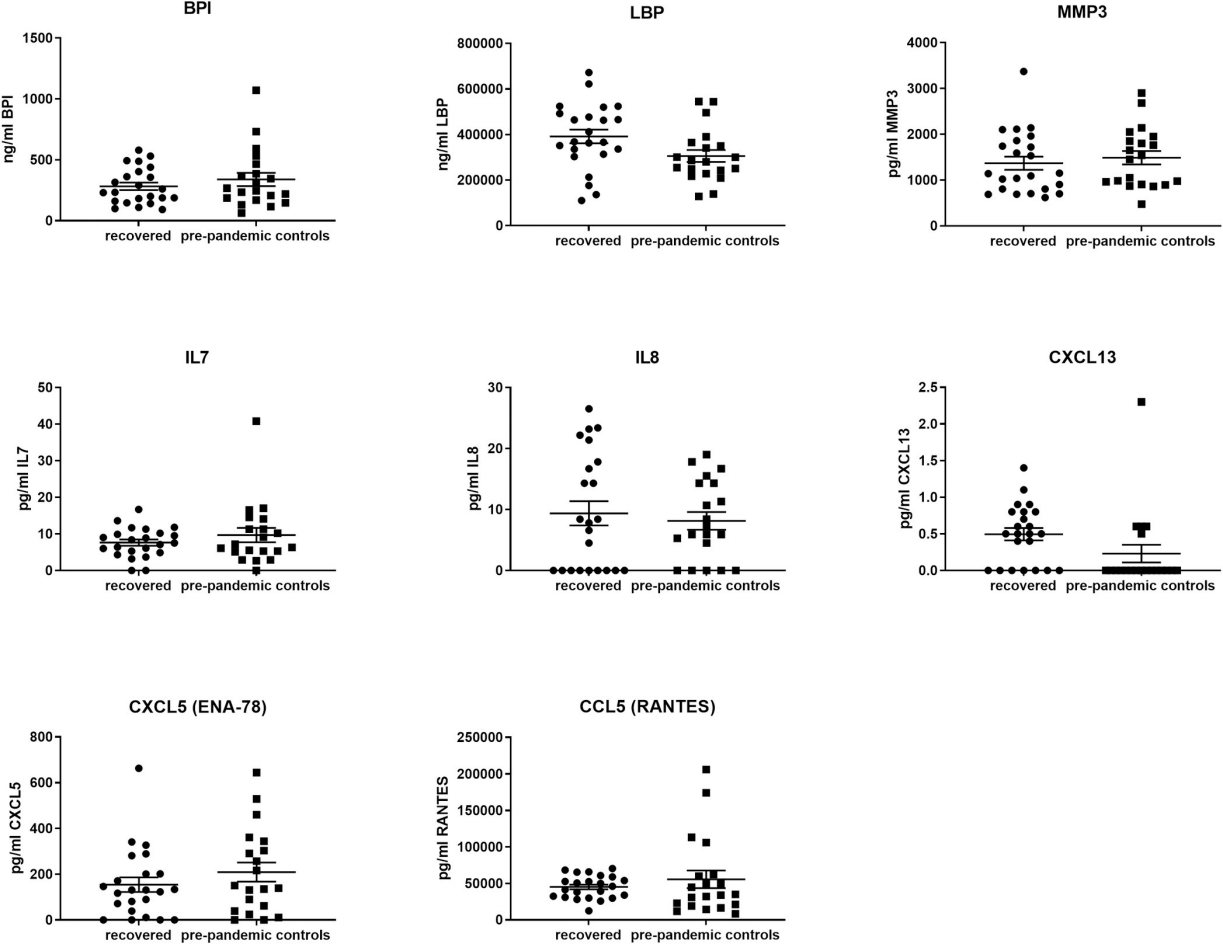
Cytokine levels of plasma donors. Cytokines, lipopolysaccharide binding protein (LBP) and bactericidal permeability increasing protein (BPI) of convalescent donors were compared to healthy control group. The following cytokines were below limit of detection: IL1β, IL1RA, IL2, IL3, IL6, IL10, IL12p40, IL15, IL21, IL22, IL23, IFNβ, IFNγ, GM-CSF, MIP1α and TNFα.

Observations by others indicate that active COVID-19 infection is associated with elevated levels of pro-inflammatory cytokines [30]. We therefore selected 24 analytes, associated with inflammatory processes or immune responses, and retrospectively compared their abundance in peripheral blood obtained from COVID-19 recovered convalescent plasma donors to a pre-pandemic control samples set (see Fig 1). We observed a non-significant increase in IL-8, LBP and CXCL13 within our donor pool compared to pre-pandemic control set values. Several parameters including IFN-γ, IL-15, MIP1a, GM-CSF, IFN-β, IL-10, IL-12p40, IL-1β, IL-21, IL-22, IL-23, IL-3, IL-6 and TNF-α, showed a negative result or were below the limit of detection (2.3–30.0 pg/mL). Levels of bactericidal/permeability increasing protein (BPI) were higher in female than in male donors. Altogether, the absence or low levels of inflammatory cytokines confirmed the recovery of the donors.

As an essential quality parameter, the quantity of the specific SARS-CoV-2 antibody (IgG) was measured. At the first appointment, a SARS-CoV-2 antibody (IgG) screening showed titers of 1:100–1:3200. The median period between screening and first donation was 5 days (range, 1–40 days). In the first 2–3 weeks of convalescent plasma manufacturing in order to facilitate rapid patient treatment, donors with low titers (< 1:400) were accepted. Subsequently, as the available donor pool increased, the titer specification of the plasma product was revised to ≥1:800, in an attempt to increase the antibody received by the patient. Within this first five weeks 45 plasmaphereses were performed with 202–829 mL (median 701 mL) resulting in 153 fresh convalescent plasma products (190–219 mL; median 200 mL) with a stability of up to five days at 2–6°C.

In order to increase the availability of convalescent plasma product for all blood groups, a program to establish a frozen plasma bank was initiated. In total, 27 apheresis were performed, resulting in 95 frozen plasma product bags (185–214 mL; median 195 mL). Plasma product was stored at -30°C. The first 13 apheresis and subsequent frozen plasma products were used for process validation. In addition to assessing the quality parameters including cell or bacterial contamination, we compared the titer of SARS-CoV-2 antibodies before and after freezing. In 12 cases (92%) the antibody titer after freezing was identical to the titer before. In one case the titer increased from 1:800 to 1:1600. However, this was likely result of a borderline antibody titer measurement. The freezing process was shown to have no negative influence on either antibody titer or antibody binding.

SARS-CoV-2 antibody titers (IgG) in the plasma ranged from 1:100 to 1:3200 and generally corresponded well to the titers in peripheral blood at the day of collection with 1:100–1:6400.

Quality parameters beside the antibody titer have been defined according to the German hemotherapy guideline [31]. The maximal concentration of residual leukocytes was 0.01 x 10^6^/mL and 16 x 10^6^/mL of platelets, which complied with the defined specifications of <1 x 10^6^/mL and <50 x 10^6^/mL, respectively. No red blood cells were detected within the final product. Randomly selected plasma samples (n = 17) were negative for bacterial contamination and no platelet aggregates or impurity were present.

**Fig 2:**
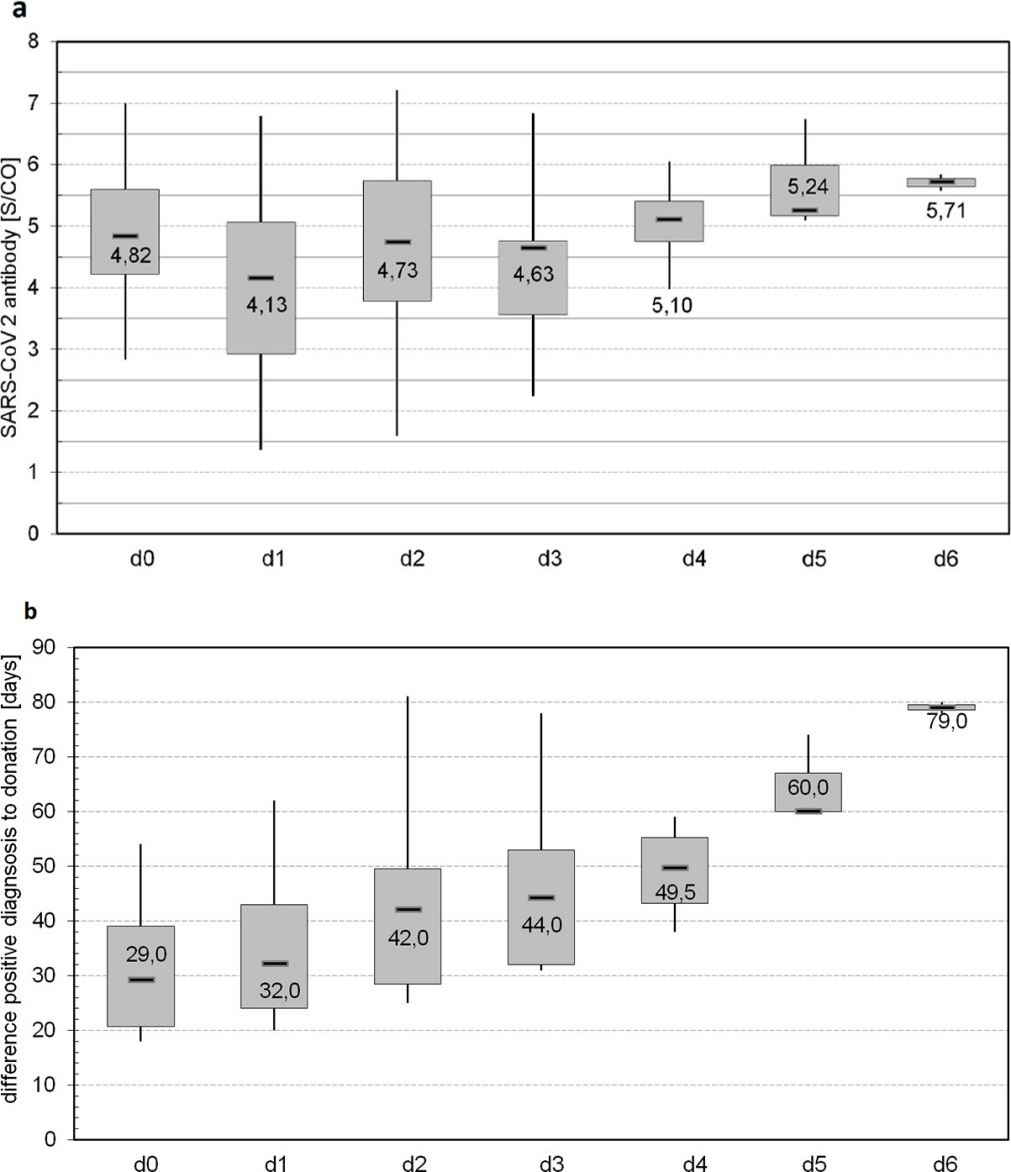
Level of SARS-CoV-2 antibody at multiple donations. a) SARS-CoV-2 antibodies [S/CO] in peripheral blood for multiple donations (≥ 2); d0 shows first appointment, d1 to d6 show donation 1 to 6. Sample size was as following: d0: 18 x, d1 and d2: 19 x, d3: 9 x, d4: 4 x, d5: 3 x, d6: 2x; b) Time [days] from positive SARS-CoV-2 NAT to multiple donations; d0 shows the period between positive test to first appointment, d1 to d6 shows period to donation 1 to 6.

The frequency of donation was dependent on multiple factors including antibody titer, availability of the donor and tolerance of apheresis procedure itself. There are data indicating a relationship between the severity of disease symptoms and the antibody titer [32]. Therefore, as part of the recruitment process, potential donors (n = 108) for our center were interviewed regarding their disease symptoms. As we have recently reported, patients with the symptoms fever, cough, fatigue or limb pain more pronounced, tended to exhibit a higher level of SARS-CoV-2 antibodies (≥ 1:1600) [33]. Furthermore, our observations suggest that the level of post-infection SARS-CoV-2 antibody may decrease overtime [34]. From the 34 convalescents 41.2% donated plasma once (n = 14), 32.4% twice (n = 11), 14.7% three times (n = 5), 2.9% each four and five times (n = 1 each) and 5.9% six times (n = 2). Thus, donors with low titers (< 1:400) participated in fewer rounds of plasmapheresis than those with higher titers. On the one hand donors with lower antibody titers should preserve their antibodies for their own protection and in addition we decided to produce plasma with a higher antibody amount. In Fig 2 the trend of antibodies of repeated donations (n = 19, ≥2 donations) is shown. For a more graduated view, the signal-to-cutoff ratio (S/CO) data instead of the titers are presented. At the day of first examination (d0) a S/CO value of 2.83–7.00 (median 4.82) was detected. This corresponds to a titer of 1:100–1:3200. From first to third donation (d1, d2: n = 19; d3: n = 9) the S/CO was not significantly different (p > 0.05). A significant (p = 0.046) S/CO was observed between d1 and d4 and ranged from 4.13 to 4.17 (median values). As levels of antibody decreased for some donors over the time, donation 4 to 6 was conducted by just 4 to 2 persons respectively, with persisting high antibody titers (1:800–1:3200) and showed therefore S/CO of 5.10–5.71 (median). Additionally, we show the period for these donors from the positive SARS-CoV-2 NAT to donation. Most of the donations (d1-d3) took place in between 20 to 78 days. Of those donors who donated plasma more than 3 times (d4-d6, 4 donors, n = 9), a constant high antibody titer was present even after 38 to 80 days. As Peterhoff et al. described there is a significant correlation of the SARS-CoV-2 antibodies titer and neutralizing antibodies with R^2^ = 0.89 (see Peterhoff et al., Fig 5 and supplementary data [26]). Thus, plasma with a high antibody titer should exhibit more neutralizing antibodies than plasma with a lower titer.

**Fig 3:**
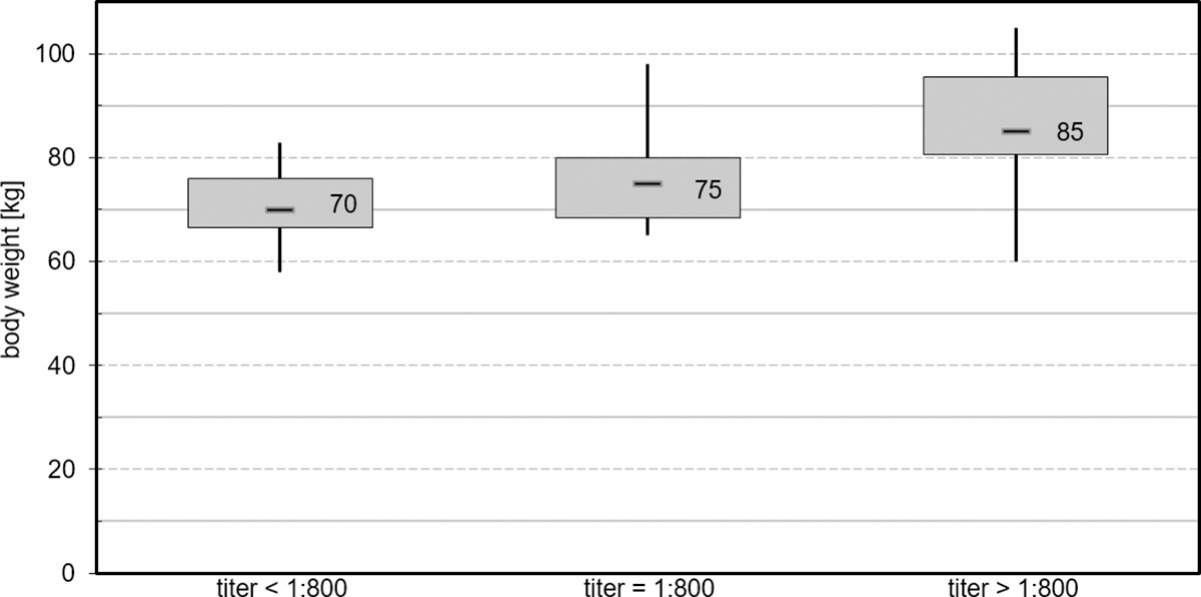
SARS-CoV-2 antibody titer in relation to donor’s body weight. Relation of patient’s body weight to SARS-CoV-2 antibody titer (IgG) divided into groups with titer <1:800 (n = 11), equal 1:800 (n = 6) and >1:800 (n = 15).

As others have reported a correlation between COVID-19 severity and patient body mass index, we investigated whether antibody level correlated to donor body weight. We observed a tendency whereby donors with a higher body weight also displayed an increased antibody titer. SARS-CoV-2 antibody titer could be grouped into three body weights; 70 kg group (< 1:800), 75 kg (1:800) and 85 kg (≥ 1:800) (median values; see Fig 3). However, this trend was not statistically significate (R^2^ = 0.154, p = 0.482).

Whilst a detailed analysis of patient outcomes is outside of the scope of this present report, of the 38 patients that have received convalescent plasma product none experienced an adverse event or serious adverse event related to convalescent plasma treatment.

Regensburg is located between two hotspots of the SARS-CoV-2 outbreak in spring 2020 within Bavaria (Counties Tirschenreuth and Rosenheim). Therefore, a high number of severe cases were referred to the University Hospital Regensburg as a maximum care facility. A number of clinical studies to assess the use of convalescent plasma as a specific COVID-19 treatment are currently being planned by several hospitals to address the needs of a potential second wave in Germany. Convalescent plasma has already been used successfully as an emergency intervention in several pandemics [35]. Nevertheless, owing to the considerable variability in study design and dosage regimen within the published COVID-19 interventional trials, it has been questioned whether it is at all possible to generate the high quality data necessary to support the use of convalescent plasma in emergency situations [36]. A key aspect to consider within such trials, and indeed in the production of therapeutic convalescent plasma outside of clinical trials, is the need to produce a scalable product of uniform quality that complies with defined specifications with a favorable safety profile. To date, no gold-standard specification exists for COVID-19 convalescent plasma. Such parameters are likely to include the presence of specific antibodies, absence of cells or bacterial contamination. Although it is expected that plasma with higher neutralizing antibody titers (≥ 1:320) would be more effective in treating COVID-19, perhaps lower thresholds might be adequate [37]. For SARS an effective antibody titer of 1:640 was reported [38]. In our study, the median antibody titer of collected plasma was 1:800 and showed a strong correlation to antibody blood titer measured before apheresis. An important observation that should be considered by treatment units that also wish to implement convalescent plasma-manufacturing program is the frequency of donation. From a pool of 34 donors, we observed that approximately 42% of donors did not attend a second apheresis visit. Indeed, donors with low antibody titers (< 1:400) were less likely to attend a second apheresis visit than those donors possessing higher titers. As there is a high correlation between antibody titer and neutralizing antibodies (R^2^ = 0.89) [26], production of plasma with higher titers was preferred. In addition, we found that relatively few donors within our pool retained a constantly high antibody level and donated more than three times. For these multiple donations, titer remained high over the observed period of 2 to 3 months.

It has been previously reported that body mass index is a relevant predictor of COVID-19 severity [39]. We therefore hypothesized that antibody titers are associated with body weight. Although no statistically significant correlation was observed, donors with a higher body weight tended to display an increased antibody titer.

When considering the manufacturing of a convalescent plasma-based product specific to COVID-19, patient safety is critical. In addition to SARS-CoV-2 antibody, a number of other donor-derived blood components and analytes including pro-inflammatory cytokines, clotting factors, bacteria and non-target natural antibodies might be present in the final convalescent plasma product. Increased levels of pro-inflammatory cytokines have been reported in COVID-19 patients [30]. Although cytokines typically have a short half-life, they represent a potentially useful biomarker for safety at the time of convalescent plasma donation [40]. Retrospective testing of cytokines in the convalescent donors reassured that the cytokine levels were not significantly different from healthy donors.

## Conclusions

In summary, this study has demonstrated that the production of highly standardized convalescent plasma (fresh and frozen) for use in the treatment of severely ill COVID-19 patients is clinically feasible. While none of the patients receiving convalescent plasma product experienced an adverse event or serious adverse event related to plasma treatment, further studies are needed in order to determine the effectiveness of this therapeutic approach. Finally, we have shown that convalescent plasma production can be rapidly and successfully implemented and that production can respond to changing demands.

## Supporting information

S1 Data (XLSX)

S2 Data (XLSX)

S3 Data (XLSX)

S1 File (ZIP)

S2 File (ZIP)

S3 File (PZFX)
